# A phenomenological model for self-initiated movement in electric fish

**DOI:** 10.1186/1471-2202-15-S1-P112

**Published:** 2014-07-21

**Authors:** Alexandre Melanson, Jorge F Mejias, James J Jun, Leonard Maler, André Longtin

**Affiliations:** 1 Department of Physics, University of Ottawa, Ottawa, Ontario, Canada, K1N 6N5; 2 Center for Neural Dynamics, University of Ottawa, Ottawa, Ontario, Canada, K1N 6N5; 3 Center for Neural Science, New York University, New York, NY 10012, USA; 4 Department of Cellular and Molecular Medicine, University of Ottawa, Ottawa, Ontario, Canada, K1H 8M5

## 

Observing behaviourally unconstrained animals can lead to simple characterization of complex behaviour. We apply this principle to infer the neural dynamics of self-initiated movement in pulse-type electric fish, *Gymnotus* sp.

Recent long-term monitoring of fish (~200 hours from 22 recording sessions, over 4 animals) in freely swimming conditions, devoid of external stimuli, reveals non-trivial structures in their pattern of electric organ discharge (EOD). Simultaneous recording of EODs and fish movement show that the EOD rate (EODR) and the activity level of the fish are bi-modally distributed, as well as highly correlated. These features thus effectively define behavioural attractor states corresponding to high and low levels of neural activity (up- and down-states, respectively). Trajectories in the EODR-activity plane consist of diffusional motion around the attractor states, interrupted by sharp transitions between states. The duration of each state is uncorrelated with that of the next up- or down-state, and is log-normally distributed with no characteristic time-scale.

Based on this data, our goal is to develop a modelling framework to better understand the neural pathway responsible for self-initiated movement. However, because the physiological parameters defining this pathway are experimentally unconstrained, it would be premature at this point to develop a detailed biophysical model of this system. There is thus a preliminary need to, instead, characterize the key features of the data from a phenomenological perspective. To address this research gap, we attempt to fit a stochastic process, with the simplest combination of dynamical components, that most closely reproduces the statistics of the data.

As a first step, we hypothesize that the first principal component of the data (EODR and activity level) follows an overdamped Brownian motion in a double-well potential with additive noise. Based on the approach of [1], we fit a 4^th^ order polynomial for the potential function by associating the stationary solution of the Fokker-Planck equation with the measured histogram. For most recording sessions, the fits appropriately reproduce the histograms, but consistently underestimates the width of the up-state potential well.

Once the potential function is determined, we generate an estimate for the noise intensity by calculating the mean escape time from small regions at the bottom of either well, and comparing it with its theoretical expression. We find, however, that this estimate is dependent on the well that was used to generate it, with all recording sessions showing a larger noise estimate for the up-state than for the down-state. Moreover, in most cases, the ratio of up- to down-state duration is underestimated by the fitted process, indicative of either too short up-states, or too long down-states compared to the data.

These findings are consistent with the above discrepancy for the width of the up-state potential well, as well as with visual inspection of the data, which shows greater variability when fish are in up-states. Taken together, these observations strongly suggest that state-dependent noise is involved in the process generating the data, either in the form of multiplicative noise, or Poisson shot noise.

## References

[B1] Deco G Martí D Ledberg A Reig R Sanchez Vives MV Effective reduced diffusion-models: a data driven approach to the analysis of neuronal dynamics PLoS Comput Biol 2009 15 12 e1000587 10.1371/journal.pcbi.1000587 19997490 PMC2778141

